# Bent spine syndrome as an initial manifestation of late-onset multiple acyl-CoA dehydrogenase deficiency: a case report and literature review

**DOI:** 10.1186/s12883-015-0380-7

**Published:** 2015-07-24

**Authors:** Yufen Peng, Min Zhu, Junjun Zheng, Yuanzhao Zhu, Xiaobing Li, Caixia Wei, Daojun Hong

**Affiliations:** Department of Neurology, The first affiliated hospital of Nanchang University, Yong Wai Zheng Street 17#, Nanchang, 330006 P.R China

**Keywords:** Late-onset multiple acyl-CoA dehydrogenase deficiency, Bent spine syndrome, Dropped head syndrome, Lipid storage myopathy, Peripheral neuropathy

## Abstract

**Background:**

Late-onset multiple acyl-CoA dehydrogenase deficiency (MADD) is an autosomal recessive inherited disease of metabolic dysfunction clinically characterized by fluctuating proximal muscle weakness, excise intolerance, and dramatic riboflavin responsiveness. Dropped head syndrome can occasionally be observed in some severe patients with late-onset MADD; however, bent spine syndrome as an initial symptom had not been reported in patients with late-onset MADD.

**Case presentation:**

A 46-year-old man lost the ability to hold his trunk upright, and had difficulty in raising his head, but he had no obvious symptoms of limb weakness. Meanwhile, he developed persistent numbness of limbs and lips around. Myopathological features and combined elevation of multiple acylcarnitines indicated that the axial myopathy might be caused by lipid storage myopathy. Cervical and lumbosacral MRI revealed a lot of abnormal signals diffusing along paravertebral muscles, while the abnormal signals almost disappeared after riboflavin treatment. Nerve conduction study indicated the patient suffering from predominantly sensory neuropathy and mildly motor neuropathy. Muscle pathology also demonstrated no typical neurogenic change, which was consistent with the electrophysiological findings. Causative mutations were found in the *ETFDH* gene.

**Conclusion:**

We report the first case of late-onset MADD with sensory neuropathy initially manifesting as bent spine syndrome and dropped head syndrome.

## Background

Bent spine syndrome (BSS) is defined as an unusual condition characterized by progressive forward flexion of the trunk [1, 2]. Selective involvement of paravertebral muscles is the most common etiology of BBS, including axial myopathy [3], motor neuron disease [4], myasthenia gravis [5], and chronic inflammatory demyelinating polyneuropathy [6].

Multiple acyl-CoA dehydrogenase deficiency (MADD) is an autosomal recessive inherited disease of fatty acid metabolism caused by deficiency of either electron transfer flavoprotein (ETF) or ETF-ubiquinone oxidoreductase (ETF:QO) [7]. Most patients with late-onset MADD can be dramatically resolved on treatment with riboflavin, so this clinical phenotype is called as riboflavin responsive MADD [8]. Besides the prominent muscular symptoms of proximal limbs, extramuscular symptoms of cardiac or gastrointestinal digestive system occasionally can be observed in some patients with late-onset MADD [9]. However, the involvement of peripheral neuropathy is seldom described in patients with late-onset MADD [10].

Some patients with late-onset MADD have weakness of the neck extensor during the disease course, even dropped head syndrome is observed in some severe cases [11]. The coexisting of bent spine syndrome and dropped head syndrome has been rarely reported in the literature [12]. Late-onset MADD was not previously described as a common cause of these two clinical features. Herein, we presented the clinical and laboratory features of a 46-year-old man, who was diagnosed as late-onset MADD with sensory neuropathy initially manifesting as bent spine syndrome and dropped head syndrome.

## Case presentation

A 46-year-old man was admitted to our clinical center with symptoms of bent spine. He initially complained of back and neck pain 3 months ago, and gradually had trouble in standing upright 2 months ago, while the symptoms would mildly alleviate when he had a rest. At the same time, he felt numbness in limbs and around lips. All of the symptoms gradually became more severe. Two weeks ago, he completely lost the ability to hold his trunk upright, and had difficulty in raising his head. The patient had no obvious symptoms of limb weakness, tremor, ataxia, and muscle cramp. He denied any family history. Neurological examination revealed a posture of bent spine and dropped head when standing up. Neck extensor strength was 2/5 (MRCS, grades 0–5); Iliopsoas muscle strength was 5-/5; proximal muscle strength of lower limb was 5/5; and distal muscle strength was 5/5. Deep tendon reflexes can not be elicited. Impairment of light touch and pain sensation were found in hands, feet, and lips around, while deep sensation was intact. Serum creatine kinase (CK) was 568 IU/L (normal 25-175 IU/L). Urinary organic acid analysis showed elevation of glutarate, 2-hydroxyglutaric acid, 3-hydroxyglutaric acid, glyceric acid, and 2-hydroxyadipic acid. Blood acylcarnitine analysis revealed a combined elevation of short- (C6), medium- (C8, C10), and long- (C14) chain acylcarnitines, which was consistent with the metabolic disturbance of MADD. Other routine blood tests were normal.

Electrophysiology showed that no sensory nerve action potentials (SNAP) were elicited in any nerves except the left ulnar nerve, which had decreased sensory conduction segmental velocities of 33 m/s across the left forearm (normal >60 m/s), and a decreased amplitude of 2.8 millivolt (mV) in the left ulnar nerves (normal >7 mV). In contrast, the results of motor nerve conduction studies were almost normal, except that compound muscle action potential (CMAP) slightly decreased in some nerves, as follows: 1.6 mV and 1.2 mV (normal >2.5 mV) in bilateral peroneal motor nerves, respectively. The electromyogram parameters of proximal and distal limb muscles were within normal limits.

**Fig. 1:**
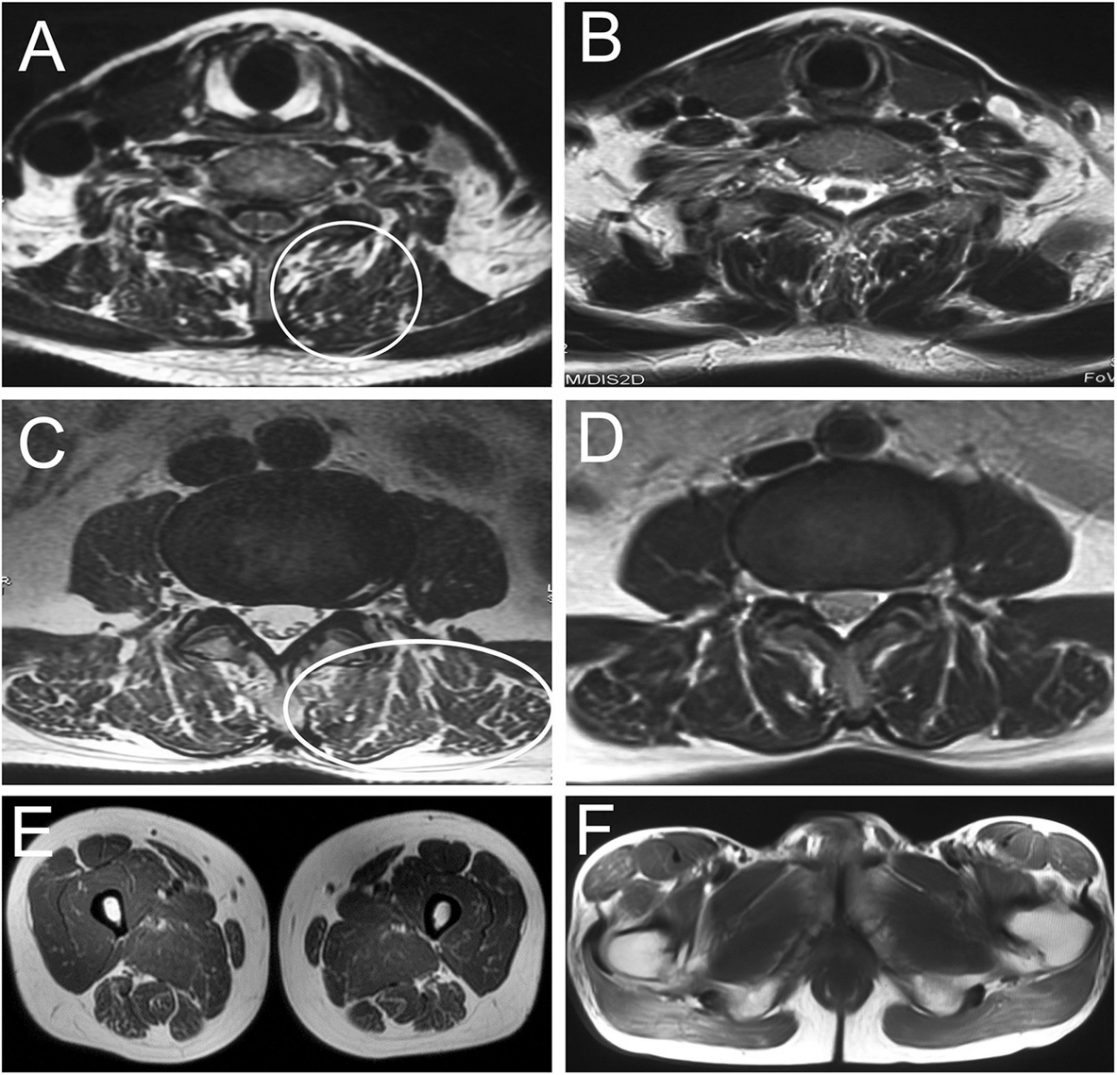
Dynamic changes of muscular MRI. Cervical MRI revealed a few abnormal signals infiltrating into cervical paravertebral muscles in T2 weighted image (**a**, circle), while the abnormal signals almost disappeared after riboflavin treatment (**b**). Lumbosacral MRI showed a lot of abnormal signals diffusing around musculi multifidus, musculi longissimus, and iliopsoas in T2 weighted images (**c**, ellipse), while the abnormal signals almost disappeared after riboflavin treatment (**d**). Lower limb MRI showed normal pattern of musculi biceps femoris, musculi quadriceps femoris (**e**), and gluteus maximus (**f**)

**Fig. 2:**
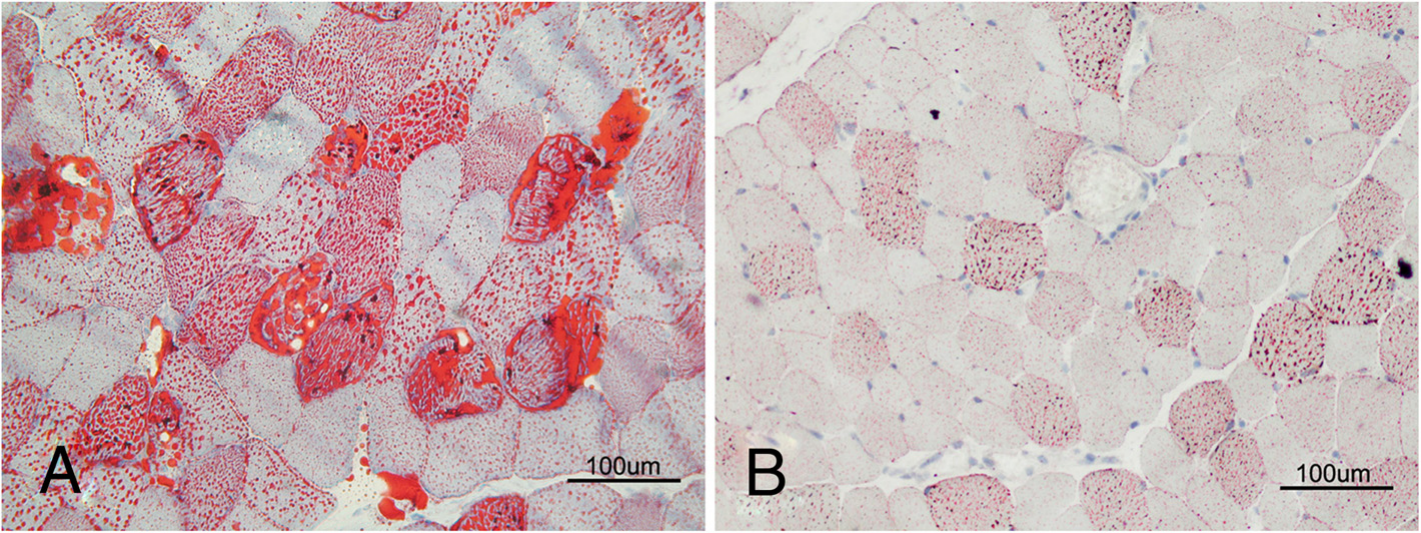
Muscular pathological features. ORO staining showed a significant increase of lipid droplets in myofibers of paravertebral muscles (**a**), but a mild increase in myofibers of quadriceps femoris (**b**)

Cervical magnetic resonance imaging (MRI) revealed a few abnormal high signals infiltrating into cervical paravertebral mucles in T1 and T2 weighted images (Fig. 1a). Lumbosacral MRI showed a lot of high signals diffusing around musculi multifidus, musculi longissimus, and iliopsoas in T1 and T2 weighted images (Fig. 1c). Lower limb MRI showed normal patterns of musculi biceps femoris, musculi quadriceps femoris (Fig. 1e), and gluteus maximus (Fig. 1f). After a written consent was signed by patient in compliance with the bioethics laws of China, open left quadriceps femoris and lumbar paravertebral muscle biopsies were performed. Hematoxylin & eosin (HE) staining revealed a lot of small vacuoles in paravertebral muscle fibers, but a few small vacuoles in quadriceps femoris. The oil red O (ORO) staining showed a significant increase of lipid droplets in paravertebral muscle fibers (Fig. 2a), but a mild increase in quadriceps femoris (Fig. 2b). No neurogenic features were observed in acid and alkaline ATPases staining.

**Fig. 3:**
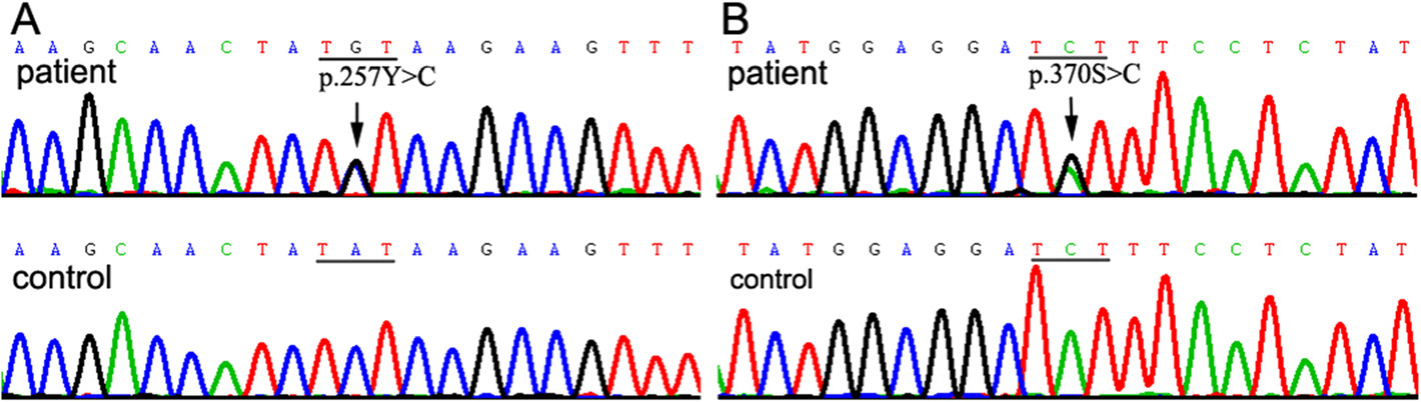
Genetic chromatogram of the *ETFDH* gene. A mutation C.770A > G (p.257Tyr > Cys) in exon 7 of the *ETFDH* gene (**a**); another mutation 920C > G (p.307Ser > Cys) in exon 8 of the *ETFDH* gene (**b**)

Genetic screening was performed in the alpha ETF (*ETFA*), beta ETF (*ETFB*), and ETF dehydrogenase (*ETFDH*) genes. A common mutation C.770A>G (p.T257C) in Chinese population was identified in exon 7 of the *ETFDH* gene (Fig. 3a). A novel mutation 920C>G (p.S307C) was found in exon 8 of the *ETFDH* gene (Fig. 3b). Substitution p.S307C is predicted to affect protein function with a score of 0.01 in Sorting Intolerant From Tolerant (SIFT) software [13] and a score of 1.0 in Polyphen2 software [14]. Blast software revealed that the mutant residue had highly evolutional conservation [15]. The novel mutation was not found in 100 health Chinese.

After administration of riboflavin (60 mg/d, for 3 weeks), the symptoms of bent spine and dropped head were completely resolved; the abnormal signals almost disappeared in musculus semispinalis cervicis (Fig. 1b), transversospinales and erector spinae (Fig. 1d); the muscle strength of neck and limbs reached 5/5. The numbness in distal limbs and around lips showed no any signs of improvement, when the patient was interviewed after 3 weeks. However, he reported a mild alleviation of numbness after persistent administration of riboflavin (30 mg/d), CoQ10 (100 mg/d), and Vitamin B12 (500 ug/d) for 6 months.

## Discussion

In this report, we described a 46-year-old patient initially presenting with bent spine syndrome and dropped head syndrome. Acylcarnitine profile showed an elevation of short-, medium- and long-chain acylcarnitines. Muscle biopsy indicated lipid storage myopathy. Because of the good response of muscle symptoms to riboflavin treatment and genetic findings, the diagnosis of multiple acyl-CoA dehydrogenase deficiency can be established [16].

Bent spine and dropped head syndromes are characterized by an abnormal flexion of the trunk and neck appearing in standing or walking position, both of which can also be called as axial myopathy [17]. Axial myopathy is mainly caused by weakness of the paravertebral muscles related to degradation of the muscular tissues, independently of the increasing age [18]. Weakness of the spinal extensor muscles can be secondary to various diseases generating pathologic changes in the anti-gravity muscles involved in trunk and neck extension. Some patients with late-onset MADD had weakness of neck extensor muscles, even presented as dropped head syndrome in some severe cases [16]. However, the severe weakness of paravertebral muscles had not been reported in patients with late-onset MADD. Our patient initially presented with bent spine syndrome, and then gradually showed dropped head syndrome. However, the patient had no obvious symptoms of limb weakness during the disease course. To our knowledge, it is the first case report of late-onset MADD presenting with bent spine syndrome as the initial symptom.

Owing to the difficulty of obtaining biopsies from paravertebral muscles, muscular radiology is becoming a helpful diagnostic tool to various axial myopathies. Computed tomography (CT) shows the commonest abnormality of axial myopathy is significant low muscle density and fatty infiltration that extends along the entire length of the paravertebral muscles [19]. MRI features of axial myopathy also have similar muscular pattern with CT changes, but the muscular MRI has a clearer contour profile than muscular CT [20]. The limb muscle MRI doesn’t show any obvious abnormalities in most patients with late-onset MADD. Occasionally, some slightly high signals in T1 and T2 weighted images were observed around musculi biceps femoris in some severe patients [21]. There is no description about the MRI pattern of paravertebral muscles in late-onset MADD. Our patient showed scattered fatty infiltration along the entire length of the paravertebral muscles, while no significant fatty signals can be observed in limb muscles. The phenomenon is consistent with the degree of myofibers with lipid droplet deposition in our patient. After administration of riboflavin, the patient had not only a complete recovery of axial muscle weakness, but also an obvious recovery of the muscular pattern in MRI. Dynamic changes of muscular MRI are a useful indicator for the diagnosis of late-onset MADD.

Nerve conduction study indicated the patient suffering from a predominantly sensory neuropathy and mildly motor neuropathy. Muscle pathology of the patient demonstrated no typical neurogenic change, which was consistent with the electrophysiological findings including prominent abnormalities of sensory nerves and slightly axonal damage of motor nerves. It demonstrated that sensory neuropathy can be one of the main manifestations in this patient with late-onset MADD [10]. Recent studies showed that zebrafish model of MADD displayed increased neural cell proliferation, abnormal glial patterning, reduced motor axon branching, as well as disorganized sensory axonal tract with hypomyelination, and decreased response to touch stimulation [22, 23]. Although the neural phenotype in MADD zebrafish model is associated with aberrant activation of the PPARG-ERK pathway and mTORC1 signaling resulting from mitochondrial dysfunction and oxidative stress, the detailed pathomechanism how mutant ETF-QO plays some roles in neurogenesis or maintenance is still unclear [24]. Treatment with riboflavin and coenzyme Q10 was quickly effective to resolve the muscle weakness, as reported by others [25], but did not resolve the sensory neuropathy in the same way. According to the fellow-up result, long-term administration of riboflavin, coenzyme Q10 and vitamin B12 may be helpful to reverse the damage of sensory nerves.

Defects of ETF:QO can cause dysfunction of the acyl-CoA dehydrogenases, which leads to defects in electron transfer generated by dehydrogenation reactions and accumulation of multiple acyl-CoAs and metabolic substrates in tissues [26]. The mutation p.257Tyr>Cys locates in flavin adenine dinucleotide (FAD) domain, and p.307Ser>Cys locates in ubiquinone (UQ) binding region. The two mutations clustered in FAD/UQ binding domain were similar to those in patients with classic late-onset MADD, but the clinical phenotype of our patient presented with great differences with classic late-onset MADD. The mutations added two cysteine residues in the junctional region between FAD domain and UQ binding domain [27]. The additional Cys residues might crosslink with the inherent Cys residue at 248 and 266 positions, which can destroy the 3D structure of ETF:QO. However, the muscular symptoms were effectively responsive to riboflavin treatment, but the peripheral sensory neuropathy was difficult to be reversible, which indicated the underlying pathogenic mechanisms of neuropathy might be different from those of muscle weakness. The genotype–phenotype correlation in this patient is needed to elucidate further.

## Conclusions

In summary, paravertebral muscles weakness can be predominant in some patients with late-onset MADD, even can present with bent spine syndrome as the initial symptom. Our case raises the awareness that bent spine syndrome and dropped head syndrome as early symptoms is advised to consider the possibility of late-onset MADD. Except for abnormalities of cardiac or gastrointestinal digestive systems, severe sensory neuropathy can be one of the extramuscular manifestations in some patients with late-onset MADD.

## Patient consent

Written informed consent was obtained from the patient for publication of this case report and any accompanying images. A copy of the written consent is available for review by the editor of this journal.

## Abbreviations

MADD: Multiple acyl-CoA dehydrogenase deficiency
BSS: Bent spine syndrome
ETF: Electron transfer flavoprotein
ETF:QO: ETF-ubiquinone oxidoreductase
CK: Creatine kinase
SNAP: Sensory nerve action potential
CAMP: Compound muscle action potential
CT: Computed tomography
SWI: Susceptibility weighted imaging
ORO: Oil red O
ETFDH: ETF dehydrogenase
FAD: Flavin adenine dinucleotide
UQ: Ubiquinone
